# General Charities

**Published:** 1916-12-16

**Authors:** 


### General Charities.

**DR. BARNARDO'S HOMES, STEPNEY CAUSEWAY, E.**—Child life is the nation's greatest asset. 2,566 children admitted since war broke out; 1,000 are war cases, mostly children of sailors and soldiers. General support for food, which gets dearer each day, for the large family of 7,480 children is appealed for.

**CHURCH ARMY.**—A special appeal is made on behalf of the Church Army Naval Auxiliary Hospital. Money and gifts in kind will be gratefully received by Prebendary Carlile, Hospital Department, 55 Bryanston Street, W.

**NATIONAL CHILDREN'S HOME AND ORPHANAGE, 104-122 CITY ROAD, E.C.**—Further support is asked for the sixteen branches, including an emigration and distribution centre at Hamilton, Canada. Not only has this institution—described recently by a leading sociologist as "an association of experts in child-saving"—given some hundreds of its "old boys" to the Navy and Army, but also it has received upwards of 200 children of our fallen fighting men, of the cost of whose maintenance the State allowance barely covers half.

### General Charities—*continued.*

**CHURCH OF ENGLAND HOMES FOR WAIFS AND STRAYS, KENNINGTON ROAD.**—Funds for the feeding, clothing, and training of over 4,700 children. Over 1,000 children of men on active service have been sheltered. 1,130 old boys are known to be serving in the Navy or Army. Six have gained commissions, and seventy-one have given their lives for their country. Head Office: Old Town Hall, Kennington Road, S.E.

**METROPOLITAN CONVALESCENT INSTITUTION, 14 VICTORIA STREET, S.W.**—A sum of £3,000 to liquidate the debt to the bankers is urgently required. Mr. W. J. Sheldrick is the Secretary.

**MENTAL AFTER-CARE ASSOCIATION, CHURCH HOUSE, DEAN'S YARD, WESTMINSTER.**—Funds to carry on and extend its work. It is the only Association of its kind in the United Kingdom. Secretary, Miss Vickers.

